# Mental health needs and accessing specialised healthcare in Mexican children with mental disorders: gender- and diagnosis-dependent differences

**DOI:** 10.1192/bjo.2023.604

**Published:** 2023-11-23

**Authors:** Lina Diaz-Castro, Kurt Hoffman, Maria Elena Marquez-Caraveo, Hector Cabello-Rangel

**Affiliations:** Direction of Epidemiological and Psychosocial Research, National Institute for Psychiatry Ramón de la Fuente Muñiz, Mexico City, Mexico; Research Center in Animal Reproduction, Autonomous University of Tlaxcala – CINVESTAV, Tlaxcala, Mexico; Research Division, Children's Psychiatric Hospital Dr Juan N. Navarro, Mexico City, Mexico; Research Department, Psychiatric Hospital Fray Bernardino Álvarez, Mexico City, Mexico

**Keywords:** Mental health services, access, child, mental disorders, specialised healthcare

## Abstract

**Background:**

Access describes factors that influence the initial contact or use of services, emphasising both the characteristics of patients and the health resources that influence the use of health services.

**Aims:**

To compare Mexican boys and girls with mental disorders, with respect to primary diagnosis, symptom onset, and seeking and accessing specialised mental health services (SMHS).

**Method:**

Longitudinal data were collected from primary caregiver-reported assessments of 397 child–caretaker dyads (child mean age 12.17 years, range 5–18 years, 63% male) that were obtained in two psychiatric hospitals specialising in child mental healthcare. Student *t*-tests and *χ*^2^-tests were applied to compare boys and girls regarding their diagnosis and variables associated with the seeking of and access to SMHS.

**Results:**

Hyperkinetic disorder was the most prevalent diagnosis in boys, whereas depressive disorder and anxiety disorder were most prevalent in girls. The mean age at symptom onset for boys was 7 years, compared with 10 years for girls. Hyperkinetic disorder had the earliest symptom onset (mean 5.9 years), followed by depressive disorder (mean 9.8 years) and anxiety disorder (mean 12 years). Delayed access to SMHS was associated with initially seeking care from a psychologist, whereas quicker access was associated with affiliation with the (now defunct) Popular Insurance, a programme that served low-income and uninsured individuals.

**Conclusions:**

Programmes aimed at children's mental health education and early intervention should consider gender- and diagnosis-related differences in symptom onset and trajectory. Access to SMHS might be improved by rapid identification by parents, educators, primary-care physicians and psychologists.

## Mental disorders in children and adolescents

International studies have shown that the prevalence of mental disorders and their contribution to the global burden of disease is higher in children and adolescents.^1^ In Mexico, mental disorders account for 5.62% of the total of the disability-adjusted life-years when both genders and all ages are considered. However, this figure rises to 11.8% when children aged 5–14 years are considered alone.^2^

Despite the high global burden of disease resulting from mental disorders in youth, there is a substantial service delivery gap between this population's urgent needs and their access to mental healthcare services, particularly in low-resource settings. Because attention has remained underprioritised,^3^ children do not receive the treatment they require, and represent an unmet need within the healthcare system.^4^

According to the epidemiological model, mental health problems are understood as ‘any alteration in health and well-being’ that require services and resources for their care,^5^ i.e. those referred to as health conditions (morbidity and mortality) are needs that motivate or induce these individuals to seek mental healthcare once these problems arise. Recent data report that the percentage of people reporting mental health needs is higher in children and women.^6^ The wide gap between health need and treatment for mental disorders in children is evidenced by the lack of access to existing interventions.^7^ Besides current research priorities for adolescents in low- and middle-income countries (LMICs) who are struggling with mental illnesses, there are other areas of vulnerability that have been identified in this population, such as suicide risk, which requires integrated services.^8^

## Access to healthcare

Access is defined as the means to approach, reach or enter a place. In the context of healthcare, this term refers to any service, provider or health institution. The term access is used to describe factors or characteristics that influence the initial contact or use of services, emphasising both the characteristics of patients and those of the health resources that influence the use of services.^9^

In Mexico, the healthcare system comprises both public and private sectors. The focus of this study is on the public sector, which provides healthcare to two main groups of people. The first group includes individuals affiliated with social security (those with formal employment), who are served by institutions like the Mexican Institute of Social Security (IMSS), the Institute of Security and Social Services of State Workers (ISSSTE), the armed forces (SEDENA and SEMAR) and the Mexican Petroleum (PEMEX). This category accounts for 48.3 million individuals, with funding coming from employers, workers and the federal government. The second group comprises individuals without social security (those without formal employment), who receive care from either the Secretary of Health, federal (SSA) or States’ (SESA) departments. Until 2019, healthcare for these 58 million people was financed either by the federal and state governments via the ‘Popular Insurance’ programme, or through out-of-pocket expenses at the point of service.^10^ The Popular Insurance programme was abolished in 2019 and replaced with the Institute of Health for Well-Being (INSABI), with the proposal to provide free healthcare and medicines.^11^

Considering that access refers to the description of factors or characteristics that influence the initial contact or use of the services, the objective of the present study was to compare Mexican boys and girls with mental disorders when seeking and accessing specialised mental health services (SMHS).

## Method

### Setting

The study took place in two psychiatric hospitals with specialised psychiatric healthcare services for children and adolescents. The first was the National Institute of Psychiatry Ramón de la Fuente Muñiz (INPRFM), an important mental health research centre with expert clinical researchers who have participated in the development of clinical practice guidelines for the detection, diagnosis and treatment of mental disorders. The clinical care services of this hospital are divided into 11 specialised clinics, one of which is the adolescent clinic. The second was the Children's Psychiatric Hospital Dr Juan N. Navarro (HPIJNN), which is the largest children's psychiatric hospital in Mexico. It provides out-patient and in-patient service delivery for low-income populations without social security, and subrogated services for adolescents in need of hospital admission who are in the social security system.^12^

### Study participants

The participants were 397 children under 18 years of age that were receiving psychiatric care at either hospital, along with their respective primary caregivers (*N* = 397).

### Design and procedure

Potential participants were selected randomly from the cohort of patients that sought care at either hospital from January 2018 to February 2020. In the out-patient care services of both hospitals, at the beginning of the working day, the consultation record was requested by the interviewers trained in the study method and supervised by the researchers L.D.-C. and M.E.M.-C. This record included data on the children, such as gender, age and diagnosis. The diagnosis was made by the psychiatrist providing care and reported in the most recent clinical note in the file. It was based on the ICD-10.^13^ Participants were randomly selected through simple sampling (one out of every three registered). These potential participants and their primary caregiver were given accurate information about the study and were invited to participate. Each girl and boy were interviewed with their primary caregiver at the same time. However, it was usually the caregiver that responded, especially with young children. Of those invited, 400 children (and their primary caregivers) agreed to participate, and three of these patients were eliminated from the study because they were older than 18 years. Written informed consent was obtained from all participants. Written consent was witnessed and formally recorded.

**Fig. 1:**
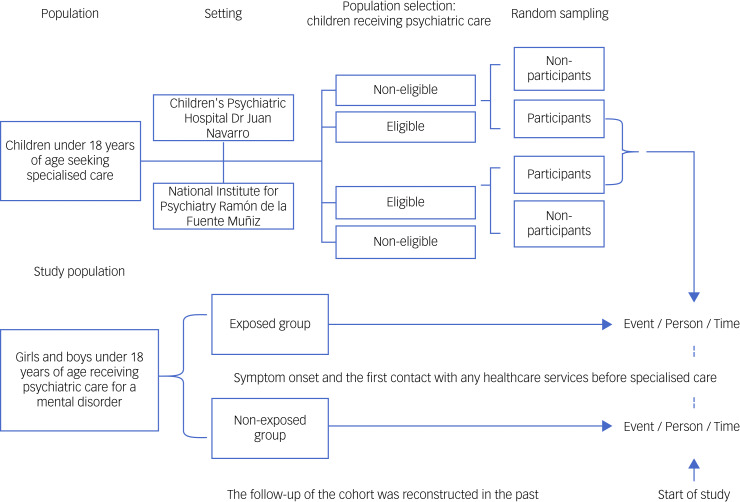
Chart review of clinical research study. The study was designed as a retrospective chart review, with the collection of data from two psychiatric hospital with specialised psychiatric healthcare services for children and adolescents (2018–2020) to identify patient demographics, symptom onset and first contact with healthcare services before specialised care.

A retrospective cohort of 397 parent–child dyads was studied, and an analytical study was carried out. Data were collected through a survey method (the application of the instrument Questionnaire Health Service Users (QHSU), described below), and organised into a database for further analysis (see Fig. 1).

The authors assert that all procedures contributing to this work comply with the ethical standards of the relevant national and institutional committees on human experimentation and with the Helsinki Declaration of 1975, as revised in 2008. All procedures involving human patients were approved by the HPIJNN and INPRFM Ethics and Research Committees (approval numbers II3/02/0917 and EP19116.0, respectively).

### Instrument

The QHSU is an *ad hoc* questionnaire that comprises three sections: section A includes sociodemographic variables (age, gender, schooling, school years completed, marital status, occupation, health insurance and family income); section B includes variables on mental healthcare needs, through eight questions about main diagnosis, comorbidity, type of healthcare facility at first contact, first adult who perceived the child's symptoms, age at onset for mental disorder, age at onset for help-seeking, first healthcare service and first SMHS; and section C encompasses data about access to treatment. Our validation of the QHSU involved a group of ten experts in the fields of child psychiatry, psychiatry, mental health services and public health. Validation was defined as ‘the process of ensuring that the questionnaire is sufficiently accurate for the purpose at hand’.^14^ To uncover potential problems in the design and application of the questionnaire, we ran a pilot study to assess the level of understanding of the respondents and the level of difficulty answering, and changes were made based on the feedback we obtained.

### Statistical analysis

Descriptive statistics for sample characteristics were carried out with measures of central tendency (mean deviation, s.d.), considering all of the variables of the QHSU. Inferential statistics were applied, with Student *t*-test for determining differences between boys and girls with respect to group means of continuous variables. *χ*^2^-tests were used to analyse categorical variables (e.g. diagnosis). Statistical significance was assumed where *P* < 0.05. An important objective of the present study was to determine how gender and diagnosis influenced seeking mental healthcare. The data were analysed with SPSS version 21 software for Windows.

## Results

### Sample characteristics

The study sample included 397 child–caregiver dyads that were receiving care at one of the SMHS. The average age of the caregivers was 41 years (s.d. = 9.2), with a mean education level of 11.7 years of study (s.d. = 3.3). Most primary caregivers (92.4%) were women, with the mother the being the main caregiver in 84.6% of cases. The main occupation of the caregiver was household activities (42.8%), followed by self-employment (28%) and formal employment (23.4%).

Of the 397 child participants, 63% were boys (*n* = 250). The mean age of the entire patient group was approximately 12 years, with an average of 6 years of schooling. Most child–caregiver dyads resided in Mexico City (83%) or the neighbouring State of Mexico (16%); 92% of patients were living with their parents. Table 1 shows some sociodemographic characteristics of the whole patient population, as well as separating by gender. Female patients were almost 3 years older and had more years of education compared with males.

**Table 1:** Sociodemographic differences by gender according to diagnosis in the sample

	All patients (*N* = 397, 100%)	Female (*n* = 147, 37%)	Male *n* = 250, 63%)	Test statistic
Age, years, mean (s.d.)	12.23 (3.7)	13.8 (3.3)	11.2 (3.5)	*t* = 7.34, *P* < 0.001
Years of education, mean (s.d.)	5.9 (3.7)	7.6 (3.4)	4.8 (3.5)	*t* = 7.53, *P* < 0.001
Place of origin				*χ*^2^ *=* 4.4, not significant
Mexico City	329 (82.9%)	116 (78.9%)	213 (85.2%)	
Mexico State	64 (16.1%)	30 (20.4%)	34 (13.6%)	
Tlaxcala	1 (0.2%)	0 (0.0%)	1 (0.4%)	
Hidalgo	2 (0.5%)	1 (0.6%)	1 (0.4%)	
Oaxaca	1 (0.2%)	0 (0.0%)	1 (0.4%)	
Schooling				*χ^2^ =* 26.6, *P* < 0.05
No study	1 (0.2%)	0 (0.0%)	1 (0.4%)	
Early childhood education	12 (3.0%)	3 (2.0%)	9 (3.6%)	
Primary education	198 (49.9%)	51 (34.7%)	147 (58.8%)	*P* < 0.001
Lower secondary education	179 (45.1%)	90 (61.2%)	89 (35.6%)	*P* < 0.001
Upper secondary education	6 (1.5%)	3 (2.0%)	3 (1.2%)	
Occupational activity				*χ^2^ =* 12.6, *P* < 0.05
Student	362 (91.2%)	125 (85.0%)	237 (94.8%)	*P* < 0.01
Inactivity owing to health issues	18 (4.5%)	12 (8.2%)	6 (2.4%)	
At-home activities (household)	5 (1.3%)	2 (1.4%)	3 (1.2%)	
Self-employed	2 (0.5%)	1 (0.6%)	1 (0.4%)	
Other	7 (1.8%)	5 (3.4%)	2 (0.8%)	
Health insurance affiliation				*χ^2^ =* 15.8, *P* < 0.01
IMSS	93 (23.4%)	39 (26.5%)	54 (21.6%)	
ISSSTE	29 (7.3%)	10 (6.8%)	19 (7.6%)	
Seguro Popular	155 (39.0%)	43 (29.2%)	112 (44.8%)	*P* < 0.01
Private	9 (2.3%)	1 (0.6%)	8 (3.2%)	
None	111 (28.0%)	54 (36.7%)	57 (22.8%)	*P* < 0.01
Family income, Mexican pesos, monthly mean (s.d.)	$6878 ($5782)	$7260 ($7652)	$6653 ($4326)	Not significant

Characteristics of the total patient sample, and of female and male patients separately. Data are expressed as mean (s.d.) or frequency (percentage of sample). Data from female and male patients were compared by a *t*-test or *χ^2^*-test/Fisher's exact test, where appropriate. *χ^2^*-statistic, Fisher's exact statistic and *t*-values are shown, along with corresponding *P*-values. IMSS, Mexican Institute of Social Security; ISSSTE, Institute of Security and Social Services of State Workers.

Regarding health insurance affiliation, 39% were affiliated with Popular Insurance, 23% with the IMSS, 7.3% with the ISSSTE, 2.3% had private insurance and 28% were uninsured. A greater proportion of male versus female patients reported using Popular Insurance, whereas a greater proportion of females versus males reported having no insurance. The mean family monthly income was $6878 (s.d. = $5782) (approximately USD344; s.d. = USD289), and this did not differ significantly between families of female and male patients.

The three most common diagnoses in this patient population were hyperkinetic disorder, depressive disorder and anxiety disorder. Girls and boys differed significantly with respect to primary diagnosis: hyperkinetic disorder was almost three times more prevalent in boys than in girls, whereas depressive disorder was almost twice as prevalent in girls compared with boys. Anxiety disorder was also much more common in girls than in boys. Approximately 60% of all patients had some comorbid psychiatric diagnosis, with anxiety disorder and depressive disorder being the most frequently observed. Comorbid anxiety disorder was significantly more common in girls than in boys. A little more than a quarter of all patients reported having some comorbid medical condition, and this did not differ significantly between girls and boys. Clinical characteristics of the 397 children according to gender are shown in Table 2.

**Table 2:** Clinical characteristics of entire children sample, and separated by gender

	All patients (*n* = 397)	Female (*n* = 147, 37%)	Male (*n* = 250, 63%)	Statistic
Diagnosis				*χ^2^ =* 77.4, *P* < 0.001
Personality disorders	2 (0.5%)	2 (1.3%)	0 (0.0)	
Anxiety disorders	31 (7.8%)	23 (15.6%)	8 (3.2%)	*P* < 0.001
Depressive disorders	135 (34%)	68 (46.2%)	67 (26.8%)	*P* < 0.001
Bipolar disorders	2 (0.5%)	2 (1.3%)	0 (0.0)	
Schizophrenia	1 (0.2%)	0 (0.0)	1 (0.4%)	
Other psychotic disorders	2 (0.5%)	0 (0.0)	2 (0.8%)	
Unspecific mental disorders	7 (1.8%)	6 (4.1%)	1 (0.4%)	
Hyperkinetic disorders	203 (51%)	39 (26.5%)	164 (65.6%)	*P* < 0.001
Dissocial behavioural disorders	4 (1.0%)	3 (2%)	1 (0.4%)	
Psychoactive substance use disorders	1 (0.2%)	0 (0.0)	1 (0.4%)	
Asperger syndrome	2 (0.5%)	0 (0.0)	2 (0.8%)	
Psychiatric comorbidity				*χ^2^ =* 21.4, *P* < 0.05
No psychiatric comorbidity	161 (40.5%)	54 (36.7%)	107 (42.8%)	
Anxiety disorders	75 (18.9%)	37 (25.1%)	38 (15.2%)	*P* < 0.05
Depressive disorders	42 (10.6%)	14 (9.5%)	28 (11.2%)	
Others	94 (23.7%)	27 (18.3%)	67 (26.8%)	
Medical comorbidity	106 (26.7%)	43 (29.3%)	63 (25.2%)	Not significant
Healthcare facility (first contact)				*χ^2^ =* 23.3, *P* < 0.001
Community mental health centre	33 (8.3%)	11 (7.4%)	22 (8.8%)	
Health centre	59 (14.9%)	17 (11.6%)	42 (16.8%)	
Family clinics/general hospital	8 (2.0%)	1 (0.7%)	7 (2.8%)	
Specialised hospital	178 (44.8%)	67 (45.6%)	111(44.4%)	
Private psychologist	63 (15.9%)	38 (25.8%)	25 (10%)	*P* < 0.001
Private child psychiatrist	44 (11.1%)	10 (6.8%)	34 (13.6%)	*P* < 0.05
None	12 (3.0%)	3 (2.0%)	9 (3.6%)	
First adult to perceive child's symptoms				*χ^2^ =* 44.7, *P* < 0.001
Mother	195 (49.1%)	92 (62.6%)	103 (41.2%)	*P* < 0.001
Teacher	90 (22.7%)	13 (8.8%)	77 (30.8%)	*P* < 0.001
Grandparents	25 (6.3%)	10 (6.8%)	15 (5.8%)	
Mother and teacher	18 (4.5%)	6 (4.1%)	12 (4.8%)	
Other family members	19 (4.8%)	11 (7.5%)	8 (3.2%)	
Both mother and father	14 (3.5%)	3 (2.0%)	11 (4.4%)	
Father	8 (2.0%)	2 (1.4%)	6 (2.4%)	
Patient	11 (2.8%)	8 (5.4%)	3 (1.2%)	*P* < 0.05
Others	17 (4.3%)	2 (1.4%)	15 (6%)	*P* < 0.05
Age at first symptoms	8.15 (4.35)	9.98 (4.32)	7.07 (3.99)	*t* = 6.64, *P* < 0.001
Age at first time seeking healthcare services	8.62 (4.52)	10.59 (4.40)	7.46 (4.19)	*t* = 6.96, *P* < 0.001
Age at first contact with healthcare services	9.32 (4.14)	11.08 (4.04)	8.28 (3.84)	*t* = 6.78, *P* < 0.001
Age at first contact with SMHS	10.62 (4.31)	12.63 (4.04)	9.44 (4.01)	*t* = 7.61, *P* < 0.001

Data are presented as frequency (percentage of sample) or mean years (s.d.). Data from female and male patients were compared by *χ^2^*-test/Fisher's exact test or *t*-test. *χ^2^*-statistic, Fisher's exact statistic and *t-*values are shown, along with corresponding *P*-values. SMHS, specialised mental health services.

### Characteristics of boys and girls when seeking and accessing SMHS

On average, patients were approximately 8 years old when they first showed symptoms, although age at symptom onset differed according to gender (Table 2) and diagnosis (Table 3). Boys showed a mean age at symptom onset of approximately 7 years old, whereas mean age at symptom onset in girls was around 10 years old (Table 2). Hyperkinetic disorder showed the earliest mean age at symptom onset (5.9 years, s.d. = 3.2), followed by depressive disorder (9.8 years, s.d. = 4.2) and anxiety disorder (12 years, s.d. = 2.9) (Table 3). Overall, the mother was most likely to be the first adult to perceive the child's symptoms. However, mothers were more likely to detect symptoms in girls compared with boys. Interestingly, the primary school teacher was almost as likely as the mother to be the first to detect symptoms in boys, and were more likely to detect symptoms in boys than in girls (Table 2).

**Table 3:** Age at symptom onset, and delay in seeking and receiving care for those symptoms

	All patients (*N* = 397)	Hyperkinetic disorder (*n* = 204)	Depressive disorder (*n* = 135)	Anxiety disorder (*n* = 31)	*t*-Statistic
Symptom onset, mean years (s.d.)	8.13 (4.34)	5.94 (3.25)	9.84 (4.24)	12.0 (2.94)	Depressive disorder versus anxiety disorder *t* = 3.4, *P* < 0.005; hyperkinetic disorder versus depressive disorder *t* = 9.0, *P* < 0.001; hyperkinetic disorder versus anxiety disorder *t* = 10.5, *P* < 0.001
Delay between symptom onset and seeking care, mean years (s.d.)	0.47 (1.34)	0.29 (1.06)	0.59 (1.43)	1.03 (2.04)	Hyperkinetic disorder versus depressive disorder *t* = 2.0, *P* < 0.05
Delay between symptom onset and first contact with healthcare professional, mean years (s.d.)	1.15 (1.81)	1.44 (1.93)	0.87 (1.55)	0.84 (1.57)	Hyperkinetic disorder versus depressive disorder *t* = 3.0, *P* < 0.005
Delay between symptom onset and first contact with specialised mental healthcare, mean years (s.d.)	2.4 (2.83)	2.58 (2.82)	2.01 (2.74)	3.06 (3.06)	Not significant
Delay between first contact with healthcare professional and first contact with specialised care, mean years (s.d.)	1.29 (2.40)	1.13 (2.27)	1.15 (2.39)	2.23 (2.83)	Anxiety disorder versus hyperkinetic disorder *t* = 2.1, *P* < 0.05

Data are expressed as mean (s.d.). Individual diagnoses were compared by *t*-tests; significant comparisons are shown along with the *t*-statistic and corresponding *P*-value.

Approximately half a year passed between symptom onset and efforts to seek care for those symptoms (0.47 years, s.d. = 1.3), and a little over a year passed between symptom onset and first contact with a healthcare professional (1.15 years, s.d. = 1.81) (Table 3). Mean age at first contact with a healthcare professional was 9.32 years (s.d. = 4.14), and this differed significantly between girls (11.08 years, s.d. = 4.04) and boys (8.28 years, s.d. = 3.84) (Table 2). Most child–caregiver dyads (45%) first sought care at a specialised hospital, followed by a private psychologist (16%), health centre (15%), private child psychiatrist (11%) or community mental health centre (8%) (Table 2). Only 3% of the child–parent dyads first sought care from SMHS. Girls were more likely than boys to seek help from a private psychologist, whereas boys were more likely than girls to have sought help from a private child psychiatrist (Table 2).

A mean of 2.4 years (s.d. = 2.83) passed between symptom onset and first contact with SMHS. The mean time between first contact with any healthcare professional and first contact with SMHS was 1.3 years (Table 3). Mean patient age at first contact with SMHS was 10.62 years (s.d. = 4.31), and girls were significantly older than boys (girls: 12.63 years, s.d. = 4.04; boys: 9.44 years, s.d. = 4.01) (Table 2).

**Fig. 2:**
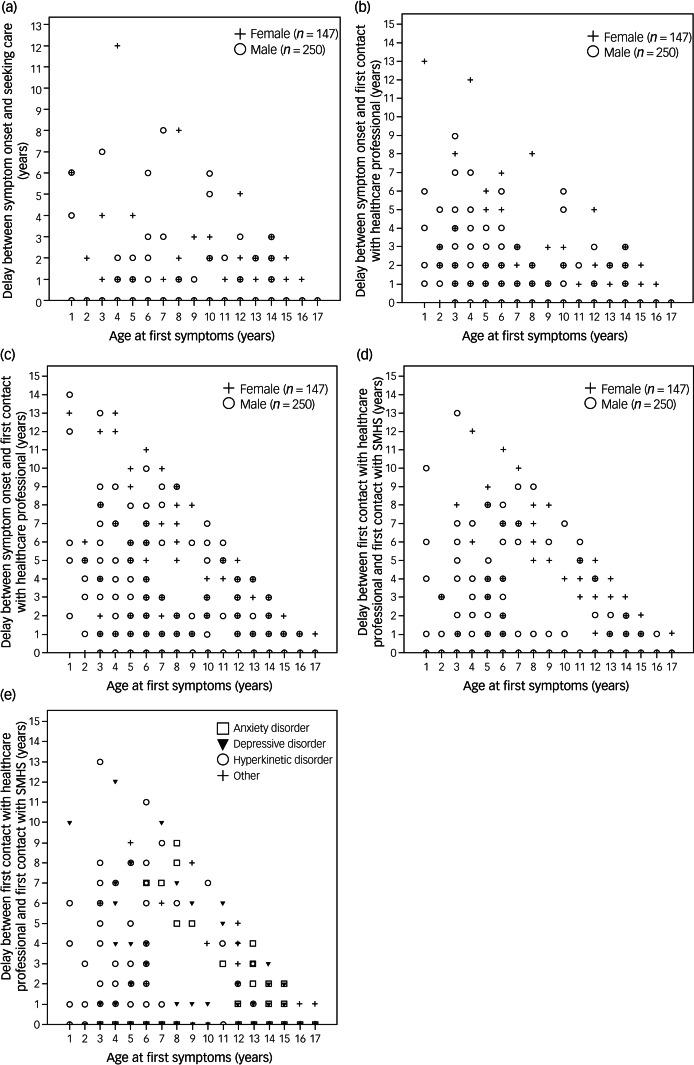
Delay between onset of psychiatric symptoms and seeking healthcare services. Estimated delay (in years) between onset of psychiatric symptoms and (a) seeking healthcare for those symptoms, (b) first contact with any healthcare professional regarding those symptoms and (c) first contact with SMHS. Panels (d) and (e) show the estimated delay between first contact with any healthcare professional and first contact with SMHS, separated by gender and by diagnosis, respectively. SMHS, specialised mental health services.

The delay between (a) symptom onset and seeking care, (b) symptom onset and first contact with a healthcare professional, and (c) first contact with any healthcare professional and first contact with SMHS, varied according to diagnosis (Table 3). Thus, considering the three main primary diagnoses, mean age at symptom onset was youngest for hyperkinetic disorder (approximately 6 years), followed by depressive disorder (10 years) and finally, anxiety disorder (12 years). The time from symptom onset to when care from a healthcare professional was first sought was shorter for hyperkinetic disorder (0.29 years) compared with depressive disorder (0.59 years) and anxiety disorder (1 year). However, the time between symptom onset and first contact with a healthcare professional was markedly longer for children with hyperkinetic disorder (1.4 years) compared with depressive disorder (0.87 years) and anxiety disorder (0.84 years). The three diagnoses did not differ significantly in the time that elapsed between symptom onset and the time of first contact with SMHS; this delay ranged from approximately 2 years for depressive disorder to approximately 3 years for anxiety disorder. The delay between first contact with any healthcare professional and first contact with SMHS was significantly longer for anxiety disorder (approximately 2 years) compared with hyperkinetic disorder (1 year) (Table 3). Figure 2 shows the delays to seek care, to receive care from any healthcare professional and to receive care from SMHS, relative to symptom onset.

The time between first contact with any healthcare professional and first contact with SMHS varied according to the healthcare provider where care was first sought (Table 4) and patient healthcare insurance affiliation (Table 5). Most notably, patients that first sought care with a private psychologist were significantly more delayed in entering SMHS (mean delay of 3 years) compared with those that first sought care at a family clinic/general hospital (mean delay of 0.62 years), specialised hospital (mean delay of 0.62 years) or a private paediatric psychiatrist (mean delay of 1.18 years). This general result was also observed when hyperkinetic disorder and depressive disorder were analysed separately (Table 4). Notably, patients with anxiety disorder were more likely to seek care with a private psychologist (36% of patients with anxiety disorder), followed by depressive disorder (21% of patients with depressive disorder) and hyperkinetic disorder (8% of patients with hyperkinetic disorder; data not shown). With regards to patient insurance affiliation, those that were affiliated with Popular Insurance entered SMHS sooner (mean delay of 0.77 years) compared with those affiliated with IMSS (mean delay of 2.13 years) (Table 5).

**Table 4:** First contact with healthcare professional and first contact with specialised mental health services, according to diagnosis

	All patients	Hyperkinetic disorder (*n* = 204)	Depressive disorder (*n* = 135)	Anxiety disorder (*n* = 31)
Health centre	**1.15 (1.67) *n* = 59**	**1.16 (1.64) *n* = 37**	0.5 (0.85) *n* = 14	1.67 (2.08) *n* = 3
Community mental health centre	2.45 (2.88) *n* = 33	2.61 (2.56) *n* = 13	2.39 (3.29) *n* = 18	No data
Family clinics/general hospital	0.62 (1.77) *n* = 8	**0.83 (2.04) *n* = 6**	0 (0) *n* = 2	No data
Specialised hospital	**0.62 (1.85) *n* = 178**	**0.53 (1.66) *n* = 102**	**0.56 (2.07) *n* = 52**	1.47 (2.44) *n* = 15
Private psychologist	2.98 (3.09) *n* = 63	3.62 (4.0) *n* = 16	2.21 (2.41) *n* = 28	3.82 (3.15) *n* = 11
Private paediatric psychiatrist	**1.18 (2.49) *n* = 44**	**1.37 (2.48) *n* = 27**	0.93 (2.71) *n* = 15	0 *n* = 1
None	**0.67 (2.31) *n* = 12**	0 (0) *n* = 2	0 (0) *n* = 6	0 *n* = 1
One-way ANOVA	F(6) = 10.3, *P* < 0.001	F(6) = 6.3, *P* < 0.001	F(6) = 3.0, *P* = 0.009	Not significant

Delay between first contact with any healthcare professional and first contact with specialised mental health services, according to type of first healthcare professional (first column) and diagnosis. Data are expressed as mean years (s.d.). One-way ANOVA compared delays according to type of first healthcare professional; ANOVA statistics are shown in the bottom row. Bold type denotes a significantly shorter delay compared with that associated with a private psychologist (*post hoc* pairwise Tukey's test). ANOVA, analysis of variance.

**Table 5:** Delay between first contact with any healthcare professional and first contact with specialised mental health services, according to type of health insurance and diagnosis

	All patients	Hyperkinetic disorder (*n* = 204)	Depressive disorder (*n* = 135)	Anxiety disorder (*n* = 31)
IMSS	2.13 (3.05) *n* = 93	1.88 (2.99) *n* = 34	2.08 (3.18) *n* = 37	2.69 (3.14) *n* = 13
ISSSTE	1.07 (2.05) *n* = 29	1.36 (2.80) *n* = 11	0.62 (1.77) *n* = 8	1.0 (0.71) *n* = 5
Seguro Popular	**0.77 (1.50) *n* = 155**	**0.75 (1.37) *n* = 106**	0.79 (1.70 *n* = 43	0.25 (0.50) *n* = 4
Private	0.78 (1.98) *n* = 9	0.33 (0.58) *n* = 3	0 (0) *n* = 2	No data
None	1.41 (2.73) *n* = 111	1.46 (2.96) *n* = 50	**0.87 (2.2) *n* = 45**	3.11 (3.25) *n* = 9
Statistic (ANOVA)	F = 5.0, d.f. = 4, *P* = 0.001	F = 3.15, d.f. = 4, *P* = 0.015	F = 3.44, d.f. = 4, *P* = 0.01	Not significant

Data are expressed as mean years (s.d.). One-way ANOVA compared delays according to type of health insurance; ANOVA statistics are shown in the bottom row. Bold type denotes a significantly shorter delay compared with that associated with IMSS (*post hoc* pairwise Tukey's test). IMSS, Mexican Institute of Social Security; ISSSTE, Institute of Security and Social Services of State Workers; ANOVA, analysis of variance.

Child–caretaker dyads were questioned about their motivation for seeking SMHS care. Considering all child–caretaker dyads, 63.7% reported that they sought care at SMHS because of non-remitting symptoms, and 14.9% said that they entered SMHS because of a worsening of symptoms (data not shown). Motivation for entering SMHS differed between the three principal diagnoses. Thus, 80.6% and 22.6% of children with anxiety disorder, 52.6% and 14.1% of children with depressive disorder, and 66.5% and 13.3% of children with hyperkinetic disorder reported the persistence or worsening of symptoms as their reason for entering SMHS (data not shown).

## Discussion

The present study reveals important characteristics of boys and girls that received specialised psychiatric care. First, there were marked differences between female and male patients with respect to psychiatric symptoms and diagnosis. Second, there were important differences between diagnoses with respect to the first detection of symptoms and the latency to receive care from any healthcare professional or from SMHS.

Delayed care from SMHS might be attributed to the healthcare service at which the child–caregiver dyad first sought care and/or to the health insurance provider to which the child–caregiver dyad was affiliated.

The data revealed differences between female and male patients with respect to the main psychiatric diagnoses. Almost two-thirds of the present patient sample were boys. Boys were three times more likely to have a primary diagnosis of hyperkinetic disorder, which was the most prevalent diagnosis in this patient sample, and mainly comprised attention–deficit hyperactivity disorder (ADHD). This finding is consistent with published literature.^15^ Depressive disorder and anxiety disorder were the second and third most prevalent disorders, and were two and five times more prevalent in girls than in boys, respectively.

With regards to the age at onset of mental disorder, our results showed a significantly earlier onset for boys (7.07 years) than girls (9.98 years); consequently, the boys in our sample were significantly younger than the girls. We also found diagnosis-dependent differences with respect to age at symptom onset: hyperkinetic disorder symptoms showed the earliest onset (5.9 years), followed by depressive disorder (9.8 years) and anxiety disorder (12 years). Our results for hyperkinetic disorder are similar in this regard to those from a recent worldwide meta-analysis that gathered data from LMICs in Africa (Ethiopia, Nigeria), the Middle East (Iraq) and Latin America (Mexico),^16^ and reported peak ages of onset of 5.5 and 9.5 years for neurodevelopmental disorders and ADHD, respectively. Interestingly, this same meta-analysis also revealed that anxiety disorder showed two peaks with regard to age at onset: 5.5 years and 15 years, also consistent with our data showing a mean age at onset of 12 years.

Considering that depressive disorder and anxiety disorder diagnoses were more prevalent in girls than in boys, and that these disorders showed a later onset than hyperkinetic disorder, it is not surprising that girls were delayed compared with boys with regards to the perception of psychiatric symptoms, seeking care and first contact with SMHS. This finding is agreement with Radez et al,^17^ who emphasised internal and external factors that may influence seeking and accessing professional care for mental health problems. Internal factors include what was previously considered (low mental health literacy) as well as embarrassment. External factors such as availability of professional help are already documented in Mexico.^18^

The delay in seeking medical care after symptom onset is an important index because early identification and intervention can mitigate disease course. Delayed care can also increase risk for future mental disorders.^19^ In Mexico, similar to what is reported in the Middle East region and in general in LMICs, there is a lack of mental healthcare workforce investment recommending the implementation of early interventions in children as a means to address stigma and delay in diagnosing and treating mental disorders.^20^ In the present patient sample, the delay between symptom onset and first contact with a healthcare professional was significantly longer for hyperkinetic disorder compared with depressive disorder. Possible explanations for this counterintuitive result might be the externalising nature of hyperkinetic disorder symptoms, and initial uncertainty regarding how and where to seek help for such symptoms.

The first adult to detect the child´s psychiatric symptoms in almost half of the cases was the mother, and a slightly lower proportion of cases were first detected by the child's schoolteacher. Nonetheless, adult detection of the child's symptoms differed between boys and girls: the mother was more likely to detect symptoms in girls, whereas the schoolteacher was more likely to detect symptoms in boys. For boys, the teacher was approximately as likely as the mother to first perceive symptoms (most likely as a result of the disruptive classroom behaviour associated with hyperkinetic disorder), whereas for girls, the mother perceived symptoms in 63% of cases, with a much lower rate of perception by the teacher and other adults (likely because of the internalising nature of depressive disorder and anxiety disorder symptoms). Regarding the parental perception of child mental health needs, a recent study in pre-schoolers (south-east region of the USA, 7% Hispanic) suggests that depressive disorders and non-externalising disorders are more likely to be perceived as a need,^21^ emphasising the multifactorial (child and parent factors) character of parental perception of need. It is well-known that parent or caregiver mental health literacy is low, and factors associated with seeking care include cultural or religious beliefs, financial barriers, limited mental health knowledge, mistrust of treatment services and stigma.^22^ Considering this result, we suggest that parents and schoolteachers would benefit from educational material regarding how psychiatric symptoms manifest in boys and girls, respectively.

For most child–caretaker dyads (approximately 45%), a specialised hospital (other than a psychiatric hospital) was the first contact with a healthcare facility. This constitutes a radical difference from what happens in high-income countries, where youth with mental health diagnoses are served mainly in school mental services (22%) or out-patient settings (21%).^23^ Lower proportions of child–caretaker dyads first sought care at a private psychologist, child psychiatrist or community mental health centre. In general, primary caregivers expressed a low degree of satisfaction with this initial care: the majority of child–caretaker dyads reported that despite receiving this care, symptoms were non-remitting or worsened, which was the primary motivation for finally seeking care with SMHS. This result reflects a lack of effective protocols and guidelines for early identification and timely and appropriate treatment,^24^ an absence in the systematisation of healthcare, as well as a lack of specialised and trained resources during the care process.

Early diagnosis and treatment by SMHS are the ideal responses to paediatric psychiatric symptoms; therefore, it is important to identify factors associated with delayed entry into SMHS. In the present sample, one such factor was the specific type of healthcare professional with which care was initially sought. Thus, if care was first sought with a private psychologist, the time that elapsed until the child–caretaker dyad entered SMHS (2–4 years) was significantly greater compared with cases in which care was first sought in a specialised hospital, a health centre or with a private paediatric psychiatrist (0.6–1.18 years). Notably, private psychologists were most often the first healthcare professional for cases of depressive disorder (21% of cases) and anxiety disorder (36% of cases), whereas fewer child–caretaker dyads chose this option in the context of hyperkinetic disorder (8% of cases). Patients that first sought care at a community mental health centre showed a similarly long delay (2–3 years) before finally entering SMHS. The present results underscore the importance of educating caretakers (and schoolteachers) on how to recognise psychiatric symptoms – especially symptoms of anxiety disorder and depressive disorder – that require SMHS, and about which healthcare services and treatment options are the most appropriate. A possible solution might be an easily accessible screening mechanism available to parents/caregivers (and perhaps schoolteachers), in which early psychiatric symptoms can be identified and recommendations given on whether symptoms require SMHS versus some other mental healthcare option (e.g. a child psychologist or community mental healthcare centre). Barriers for the integration of services into primary healthcare in LMICs are well-documented and demand greater investment on primary healthcare strengthening, capacity building for health providers and higher levels of support for the social needs of the population.^25^ Interventions improving child mental health outcomes according to Preferred Reporting Items for Systematic Reviews and Meta-Analyses (PRISMA) guidelines revealed only one study in Mexico at a community level, and none in specialised services.^26^

Another factor associated with delayed entry into SMHS was specific affiliation with a health insurance provider. Our data showed that 30% of the child–caretaker dyads that sought care with SMHS had social security (IMSS and ISSSTE), another 30% did not have healthcare insurance and the remaining 40% were affiliated with Popular Insurance (a programme that protected people without social security). Child–caretaker dyads that were affiliated with IMSS showed a significantly longer delay entering SMHS compared with those affiliated with Popular Insurance. As explained earlier, IMSS is a federal entity that provides healthcare and other benefits to citizens that are formally employed. Care providers within the IMSS system include specialised hospitals located throughout Mexico. By contrast, Popular Insurance was a programme designed to provide health insurance to those citizens that were not covered by other federal programmes, such as those who were not formally employed. Consequently, in the present sample, average family monthly income of child–caregiver dyads that were affiliated with IMSS was almost twice that of those affiliated with Popular Insurance (data not shown). Child–caretaker dyads affiliated with Popular Insurance may have entered SMHS sooner because one of the SMHS hospitals (the HPIJNN) provides healthcare specifically for low-income populations without social security.^12^ Moreover, a significantly greater proportion of those affiliated with IMSS (28%) initially sought care with a private psychologist compared with those affiliated with Popular Insurance (7%; data not shown). This result underscores the need for mechanisms by which those affiliated with IMSS can be expeditiously channelled into SMHS and the necessity of including mental health in the national health insurances schemes, as has been suggested for other LMICs similar to Mexico.^27^

This study is limited to the characteristics of boys and girls who received specialised psychiatric care. The study identifies important differences between male and female patients with respect to psychiatric symptoms and diagnoses, as well as diagnosis-dependent differences in the onset of symptoms. The study also identifies factors associated with delayed entry into SMHS, such as the specific type of healthcare professional with which care was initially sought and the delay in seeking medical care after symptom onset. However, the study does not provide information about the effectiveness of the specialised psychiatric care received or the long-term outcomes of the patients. The study is also limited to the patient sample used and does not include patients who did not receive specialised psychiatric care. Finally, the study is limited to the context of Mexico and may not be generalisable to other countries.

## Data Availability

The data that support the findings of this study are available on request from the first author, L.D.-C. The data are not publicly available because they contain information that could compromise the privacy and confidentiality of research participants.
